# Supplementary material for: Identification of Novel Therapeutic Targets for Polyglutamine Diseases That Target Mitochondrial Fragmentation
Source: Int J Mol Sci. 2021 Dec 14;22(24):13447. doi: 10.3390/ijms222413447 (PMC8703635; doi:10.3390/ijms222413447)
Supplement: Supplementary file 1 [file ijms-22-13447-s001.zip › ijms-1459979-supplementary.pdf]

Supplementary Information for:

Identification of novel therapeutic targets for polyglutamine diseases that target mitochondrial fragmentation

Annika Traa[1,2]*, Emily Machiela[3*], Paige D. Rudich[1,2], Sonja K. Soo[1,2], Megan M. Senchuk[3], Jeremy M. Van Raamsdonk[1,2,3,4,5]

[1]Department of Neurology and Neurosurgery, McGill University, Montreal, Quebec, H4A 3J1, Canada
[2]Metabolic Disorders and Complications Program, and Brain Repair and Integrative Neuroscience Program, Research Institute of the McGill University Health Centre, Montreal, Quebec, H4A 3J1, Canada
[3]Laboratory of Aging and Neurodegenerative Disease, Center for Neurodegenerative Science, Van Andel Research Institute, Grand Rapids MI 49503, USA
[4]Division of Experimental Medicine, Department of Medicine, McGill University, Montreal, Quebec, Canada
[5]Department of Genetics, Harvard Medical School, Boston MA 02115, USA

*these authors contributed equally to this work

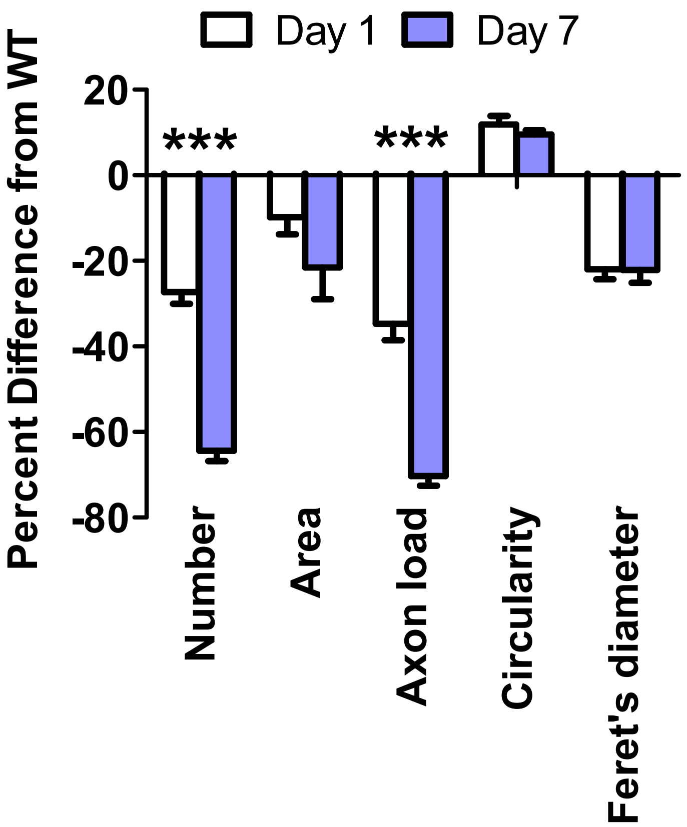

Figure S1. Differences in mitochondrial morphology in neuronal model of polyglutamine toxicity worsen with age. This figure compares the percentage change in measurements of mitochondrial morphology in Neur-67Q worms relative to wild-type worms at day 1 and day 7 of adulthood (data from **Figure 1**). The decrease in mitochondrial number and axonal mitochondrial load exhibited significantly greater deficit at day 7 of adulthood compared to day 1 of adulthood. Statistical significance was assessed using a two-way ANOVA with Bonferroni's multiple comparison test. Error bar indicate SEM. ***$p<0.001$.

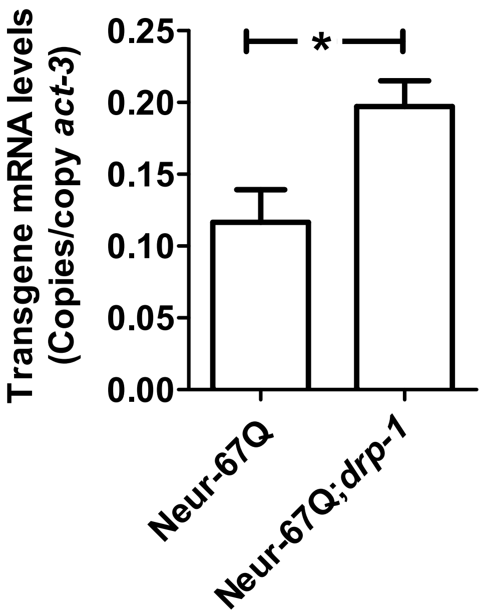

Figure S2. Deletion of *drp-1* results in increased expression of the polyglutamine transgene. mRNA was isolated from day 1 pre-fertile young adult worms. Primers were designed to target YFP. In Neur-67Q worms, deletion of *drp-1* significantly increased mRNA expression levels of the polyglutamine transgene. Bars indicate mean value of three biological replicates. Statistical significance was assessed using a t-test. Error bars indicate SEM. *$p<0.05$.

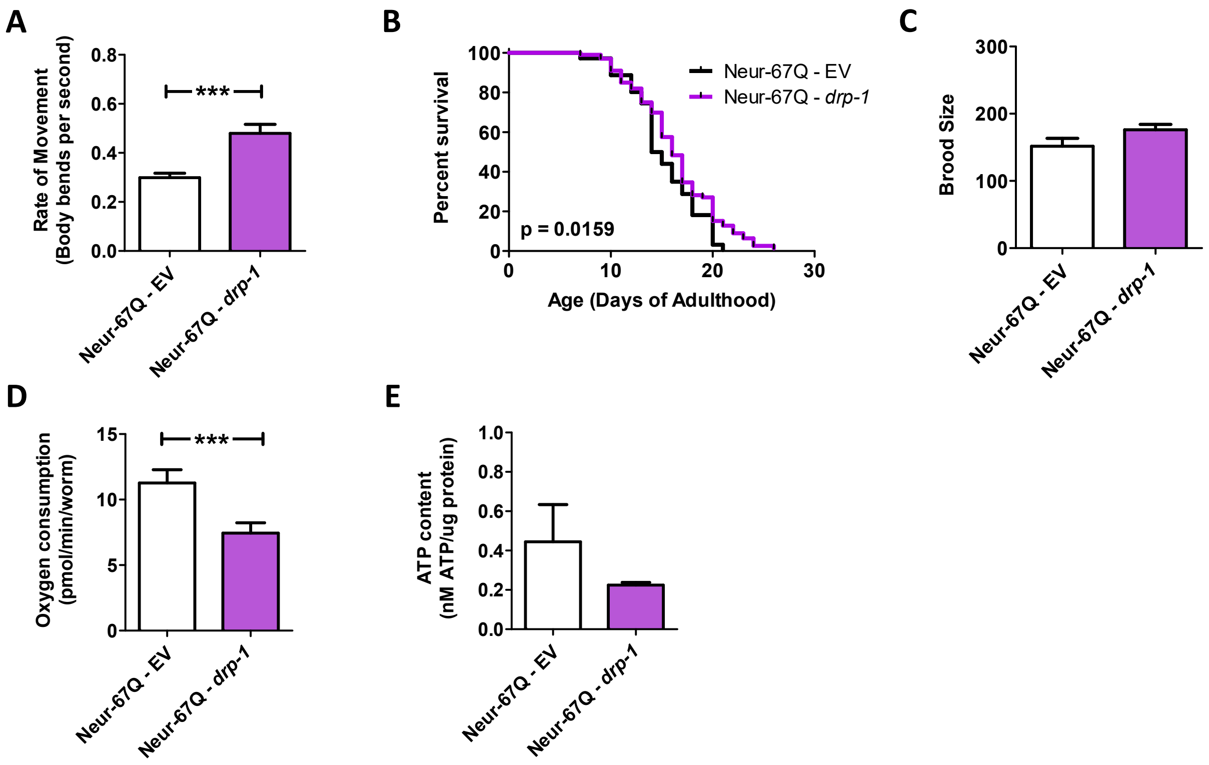

Figure S3. Decreasing mitochondrial fission through *drp-1* RNAi increases movement and lifespan in neuronal models of polyglutamine toxicity. Neur-67Q worms were crossed to *sid-1;unc-119p::sid-1* worms to allow for RNAi only in neurons. *drp-1* RNAi increases movement rates in Neur-67Q worms (**A**) and results in a small but significant increase in lifespan in Neur-67Q worms (**B**). *drp-1* RNAi has no effect on fertility in Neur-67Q worms (**C**). *drp-1* RNAi significantly reduced oxygen consumption in Neur-67Q worms (**D**) and resulted in a trend towards decreased ATP levels (**E**). A minimum of three biological replicates were performed. Bars indicate the mean value. Significance was assessed using a t-test (A,C,D,E) or log-rank test (B). Error bars indicate SEM. ****p*<0.001.

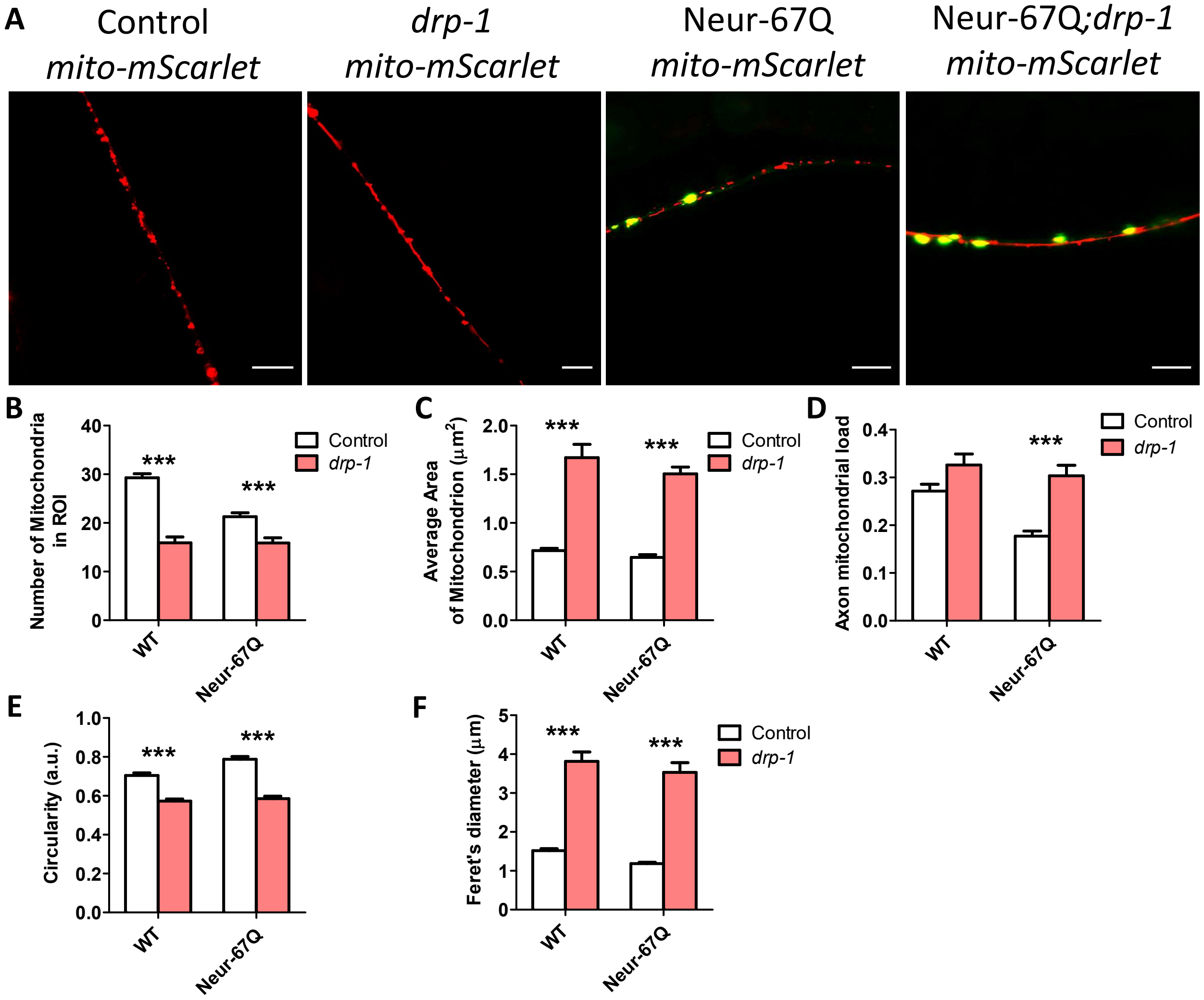

Figure S4. Disruption of *drp-1* markedly elongates mitochondria in neurons. Deletion of *drp-1* decreased mitochondrial fragmentation in Neur-67Q and control worms. Representative images of Neur-67Q worms and control worms in wild-type and *drp-1* deletion background (**A**). Scale bar indicates 10 μM. Quantification of mitochondrial morphology revealed that *drp-1* deletion significantly decreased mitochondrial number (**B**), increased mitochondrial area (**C**), increased axonal mitochondrial load (**D**), decreased mitochondrial circularity (**E**), and increased the Feret's diameter of the mitochondria (**F**) in both Neur-67Q and control worms. Control worms are *rab-3p::tomm-20::mScarlet.* For panels B-F, "Control" refers to worms in a wild-type background with normal expression of *drp-1* (either wild-type or Neur-67Q worms). Control data is from Figure 1 and is shown to facilitate direct comparison of the effects of *drp-1* deletion. Three biological replicates were performed. Statistical significance was assessed using a two-way ANOVA with Bonferroni posttest. Error bars indicate SEM. ***p*<0.01, ****p*<0.001.

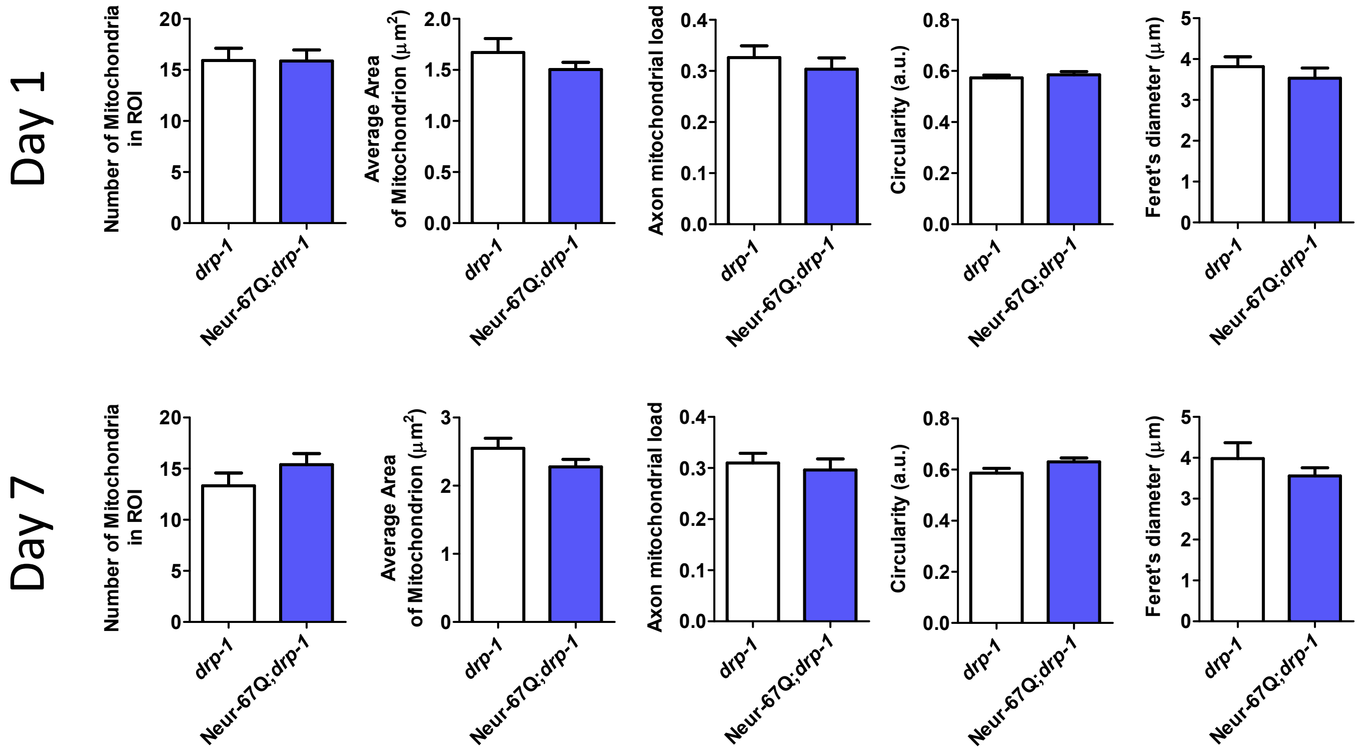

Figure S5. Expression of disease-length polyglutamine in neurons does not affect mitochondrial morphology in *drp-1* mutant background. This figure is displaying the data from **Figure 4** and **Figure S4** in such a way to directly compare *drp-1* worms to Neur-67Q;*drp-1* worms. There were no significant differences in any measure of mitochondrial morphology between *drp-1* and Neur-67Q;*drp-1* worms at day 1 (top) or day 7 (bottom) of adulthood. Thus, disruption of *drp-1* completely eliminates differences in mitochondrial morphology between Neur-67Q and wild-type worms. Three biological replicates were performed. Statistical significance was assessed using a t-test. Error bars indicate SEM.

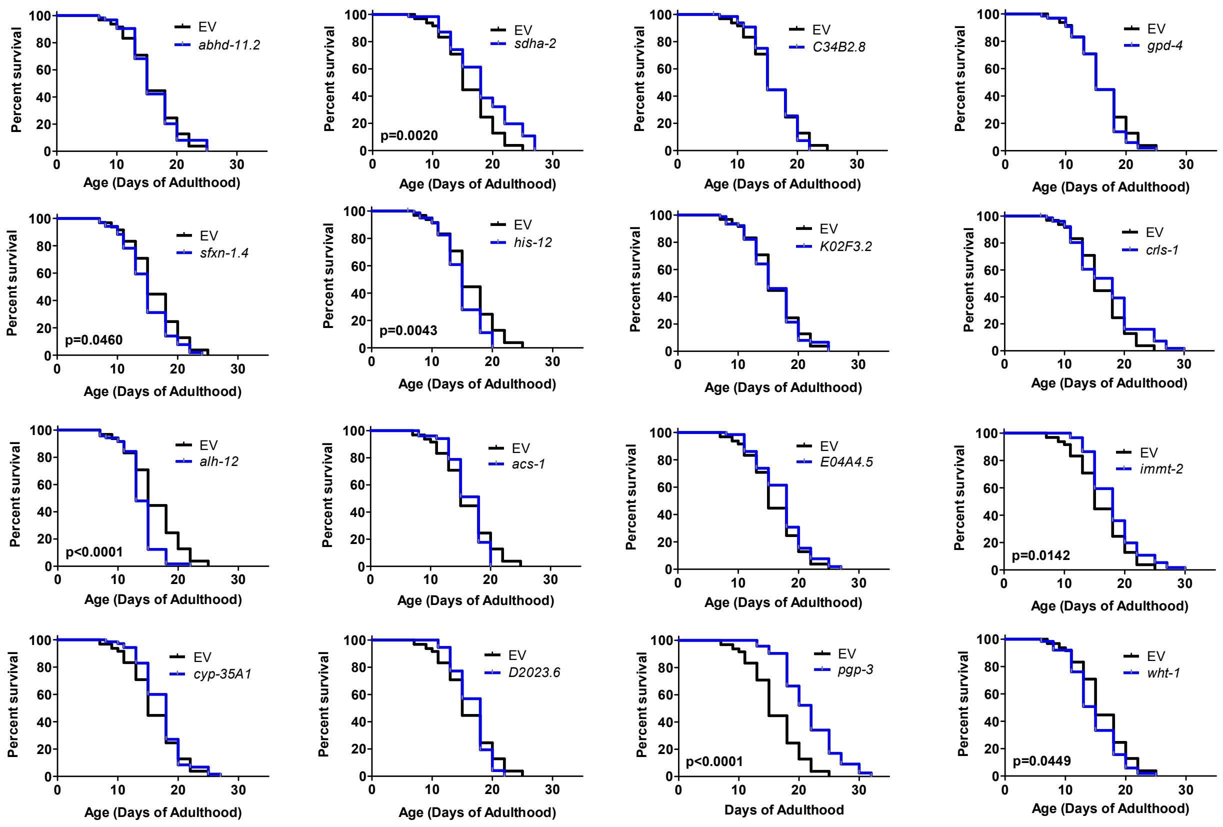

Figure S6. Neuron-specific knockdown of *sdha-2, immt-2* and *pgp-3* extends lifespan in a wild-type background. *unc-119p::sid-1;sid-1* control worms were treated with RNAi clones that decrease mitochondrial fragmentation and improved movement in Neur-67Q worms. Only three of the sixteen RNAi clones that improved movement in Neur-67Q worms resulted in increased lifespan in control worms. Three biological replicates were performed. Log-rank test was used to assess significance.

Table S1. Comparison of neuronal and body wall muscle models of polyglutamine toxicity. Data from BW-Htt74Q worms is from Machiela *et al.* 2021, *Aging and Disease*, PMID: 34631219).

	Wild-type	Neur-67Q model	BW-Htt74Q model
**Promoter**		*rgef-1*	*unc-54*
**Huntingtin sequence**		None	Exon 1
**Glutamines**		67	74
**Fluorescent tag**		YFP	GFP
**Expression**		All neurons	Body wall muscle
**Mitochondrial number**		Decreased	Increased
**Mitochondrial area**		Unchanged	Decreased
**Mitochondria circularity**		Increased	Increased
**Oxygen consumption**		Increased	Unchanged
**ATP levels**		Decreased	Unchanged
**Movement**		Decreased	Decreased
**Lifespan**		Decreased	Decreased
**Brood size**		Decreased	Decreased
**Development time**		Increased	Unchanged
**Effect of *drp-1* deletion**			
**Mitochondrial morphology**	Elongated	Elongated	Unchanged
**Oxygen consumption**	Unchanged	Decreased	Unchanged
**ATP levels**	Decreased	Decreased	Decreased
**Movement**	Unchanged	Increased	Decreased
**Lifespan**	Unchanged	Increased	Decreased
**Brood size**	Decreased	Unchanged	Decreased
**Development time**	Increased	Unchanged	Increased
**Effect of *drp-1* RNAi**			
**Oxygen consumption**	Unchanged	Decreased	Unchanged
**ATP levels**	Decreased	Decreased	Decreased
**Movement**	Increased	Increased	Unchanged
**Lifespan**	Unchanged	Increased	Increased
**Brood size**	Decreased	Unchanged	Decreased
